# Evaluation of the diagnostic and prognostic clinical values of circulating tumor DNA and cell-free DNA in pancreatic malignancies: a comprehensive meta-analysis

**DOI:** 10.3389/fonc.2024.1382369

**Published:** 2024-06-25

**Authors:** Mehmet Emin Arayici, Abdullah İnal, Yasemin Basbinar, Nur Olgun

**Affiliations:** ^1^ Department of Biostatistics and Medical Informatics, Faculty of Medicine, Dokuz Eylül University, İzmir, Türkiye; ^2^ Department of Public Health, Faculty of Medicine, Dokuz Eylül University, İzmir, Türkiye; ^3^ Department of General Surgery, Faculty of Medicine, İzmir Democracy University, İzmir, Türkiye; ^4^ Department of Translational Oncology, Institute of Oncology, Dokuz Eylül University, İzmir, Türkiye; ^5^ Department of Clinical Oncology, Institute of Oncology, Dokuz Eylül University, İzmir, Türkiye

**Keywords:** CtDNA, cfDNA, pancreatic malignancy, meta-analysis, clinical value

## Abstract

**Background:**

The diagnostic and prognostic clinical value of circulating tumor DNA (ctDNA) and cell-free DNA (cfDNA) in pancreatic malignancies are unclear. Herein, we aimed to perform a meta-analysis to evaluate ctDNA and cfDNA as potential diagnostic and prognostic biomarkers.

**Methods:**

PRISMA reporting guidelines were followed closely for conducting the current meta-analysis. The PubMed/Medline, Scopus, and Web of Science (WoS) databases were scanned in detail to identify eligible papers for the study. A quality assessment was performed in accordance with the REMARK criteria. The risk ratios (RRs) of the diagnostic accuracy of ctDNA compared to that of carbohydrate antigen 19.9 (CA 19.9) in all disease stages and the hazard ratios (HRs) of the prognostic role of ctDNA in overall survival (OS) were calculated with 95% confidence intervals (CIs).

**Results:**

A total of 18 papers were evaluated to assess the diagnostic accuracy and prognostic value of biomarkers related to pancreatic malignancies. The pooled analysis indicated that CA19.9 provides greater diagnostic accuracy across all disease stages than ctDNA or cfDNA (RR = 0.64, 95% CI: 0.50–0.82, *p* < 0.001). Additionally, in a secondary analysis focusing on prognosis, patients who were ctDNA-positive were found to have significantly worse OS (HR = 2.00, 95% CI: 1.51–2.66, *p* < 0.001).

**Conclusion:**

The findings of this meta-analysis demonstrated that CA19-9 still has greater diagnostic accuracy across all disease stages than KRAS mutations in ctDNA or cfDNA. Nonetheless, the presence of detectable levels of ctDNA was associated with worse patient outcomes regarding OS. There is a growing need for further research on this topic.

**Systematic review registration:**

https://doi.org/10.37766/inplasy2023.12.0092, identifier INPLASY2023120092.

## 1 Introduction

It is well known that tumors arising in the pancreas constitute one of the most prevalent malignancies affecting the upper gastrointestinal (GI) tract, following esophageal and gastric cancers. Moreover, it is one of the most difficult malignancies to manage due to its rapid progression and resistance to conventional treatments (1–3). The early stages of the disease typically show minimal or no symptoms (1, 2). Despite the introduction of new therapeutic approaches, the overall prognosis is generally unfavorable due to the aggressive nature of the tumor and remarkable recurrence rates. This emphasizes the importance of the crucial need for developing more effective diagnostic methods (3–6).

In clinical diagnostics for upper gastrointestinal malignancies, traditional serum protein markers such as carcinoembryonic antigen (CEA) and carbohydrate antigen 19.9 (CA19.9) are increasingly being recognized. However, these markers have important limitations, especially in terms of sensitivity and specificity (4, 7). Therefore, circulating tumor DNA (ctDNA) and cell-free DNA (cfDNA) can be used to detect the dynamic and genomic alterations associated with pancreatic malignancies (5, 6).

Liquid biopsy techniques such as ctDNA and cfDNA are promising methods for improving diagnostic accuracy and treatment strategies (8). A growing evidence base emphasizes the superiority of liquid biopsies compared to traditional tissue biopsies. Their noninvasive nature, increased safety, and improved ability to manage the complexities of intratumor heterogeneity are well documented (5, 6, 9). Additionally, it has been suggested that ctDNA provides faster and more accurate predictions than radiological imaging (6, 9). Furthermore, these studies also highlight the ability of ctDNA to detect real-time tumor dynamics with advances in technology.

Previous reports of primary research on the diagnostic accuracy of ctDNA and CA 19.9 have presented various contradictory findings. Several studies have proposed that CA 19.9 has notably greater diagnostic accuracy than ctDNA (10, 11). These studies also suggest that CA 19.9 may be more reliable. It has been well validated in many patient groups and is commonly used in clinical practice. Conversely, other studies propose no or negligible diagnostic difference between them (12, 13). Notably, numerous factors may have contributed to these inconsistent results, including the methodologies of the studies, the specific patient populations in the studies, and the technological platforms. Therefore, it is important and necessary to conduct more robust large-scale comparative studies, especially meta-analyses, to assess the current landscape associated with these biomarkers.

Herein, we performed a comprehensive meta-analysis to evaluate ctDNA and cfDNA as potential diagnostic and prognostic biomarkers for pancreatic malignancies.

## 2 Methods

### 2.1 Literature search and search strategy

Preferred Reporting Items for Systematic Reviews and Meta-Analysis (PRISMA) reporting guidelines were followed closely for documenting the current meta-analysis (14, 15). To validate adherence to established guidelines, the PRISMA checklist has been incorporated into **Supplementary Table S1**, serving as a key tool to ascertain compliance. The protocol for the meta-analysis was registered at the International Platform of Registered Systematic Review and Meta-analysis Protocols (INPLASY) and assigned the registration number INPLASY2023120092. The PubMed/Medline, Scopus, and Web of Science (WoS) databases were scanned in detail to identify eligible papers for the meta-analysis. The relevant databases were searched until December 2023, and there was an update in January 2024. Only articles published in English and with full-text access were evaluated, and papers published in other languages were not included in the initial search. The following main keyword combinations were used to develop the search strategy: ((Pancreatic[Title/Abstract]) AND (((((Carcinoma[Title/Abstract]) OR (cancer[Title/Abstract])) OR (neoplasm[Title/Abstract])) OR (tumor[Title/Abstract])) OR (tumour[Title/Abstract]))) AND ((circulating cfDNA[Title/Abstract]) OR (circulating ctDNA[Title/Abstract])). Medical Subject Headings (MeSH) and text terms were used to associate keywords, which were then combined using Boolean operators (AND/OR). The search method was developed in PubMed/Medline and then modified for use with additional databases (WoS and Scopus). The search procedures and related keyword combinations are documented in **Supplementary Table S2**.

The PICOS framework was as follows:

Population: This study involved patients who were diagnosed with pancreatic malignancies.
Intervention: The intervention under consideration was the presence of ctDNA and cfDNA.
Comparison: The comparison included comparing the presence of ctDNA or cfDNA with CA 19.9 for diagnostic accuracy and assessing the presence of ctDNA for overall survival.
Outcomes: OS in the presence of ctDNA and the diagnostic accuracy of ctDNA and cfDNA.
Study design: Cohort, case−control.

### 2.2 Study selection and inclusion-exclusion criteria

Original papers that provided reports related to ctDNA-cfDNA in pancreatic cancer were included in this meta-analysis. Two researchers independently assessed the titles and abstracts of the studies included in the initial review. Papers that were considered irrelevant based on their title and abstract were quickly omitted from the analysis. Disagreements were settled by discussion until consensus was achieved. Possible duplications were isolated after downloading potential studies that could be included in the study. The first two authors summarized the findings provided in the initial investigations after separating potential duplicate papers. Then, the studies were processed into a preprepared Microsoft Excel spreadsheet.

Studies had to meet the following selection criteria: i) the study population should include patients with pancreatic malignancies; ii) at least one of these patient groups must have had ctDNA or cfDNA analysis; and iii) the study population should contain information about the diagnostic, prognostic or predictive values of ctDNA and cfDNA. Studies that did not meet the related criteria were omitted from the meta-analysis. Moreover, conference reports, unpublished preprints, reviews, or case reports were not included in this meta-analysis.

### 2.3 Data extraction, acquisition, and quality assessment

All the data were processed independently by the first two authors (MEA and AI) according to a preprepared protocol. The following features of the studies were reviewed: first author name, year of publication, number of cases and controls, type and time of sampling, marker(s) or gene(s) of interest, cfDNA isolation, and sequencing method. In addition, the hazard ratios (HRs) for overall survival (OS) with 95% confidence intervals (CIs) were also calculated. All data obtained in the study were cross-checked by two authors via a standard spreadsheet to reach a consensus.

Quality assessment was performed in accordance with the REporting recommendations for Tumor MARKer Prognostic Studies (REMARK) criteria and is presented in **Supplementary Table S3** (16). Each study can be given one point out of seven criteria. In the case of uncertainty, it was scored by giving half a point. Studies scoring ≥ 5.5 were included in the meta-analysis for further analysis.

### 2.4 Statistical analysis

Previous studies evaluating ctDNA, cfDNA, and CA 19.9 levels, as well as the prognostic role of ctDNA in OS, were considered in primary and secondary meta-analyses. The diagnostic accuracy of ctDNA or cfDNA compared to CA 19.9 in all disease stages was included in the primary meta-analysis. The prognostic role of ctDNA in OS was also included in the secondary meta-analysis. The risk ratios (RRs) were calculated for the diagnostic accuracy of ctDNA/cfDNA compared to CA 19.9 In the evaluation of the prognostic role of ctDNA, data regarding the relationship between the presence of ctDNA and OS were pooled and reported as hazard ratios (HRs). The outcomes of the meta-analysis are graphically illustrated via forest plots. Cochran’s Q test and I^2^ statistics were calculated to measure the statistical heterogeneity between studies in performing all the meta-analyses. The presence of significant heterogeneity was evaluated as follows: an I2 greater than 50% and a *p* value < 0.05 according to Cochran’s Q test (17). When it was determined that the outputs were heterogeneous, the analyses were carried out using the random effects model. Otherwise, the fixed-effects model was used to conduct the meta-analyses.

To demonstrate the robustness of the outcomes in this meta-analysis, sensitivity analyses were performed. The approach involved reassessing the effect size (ES)—a measure used to quantify the strength of the relationship or association—by sequentially omitting each study from the pooled meta-analysis. By removing one study at a time and recalculating the pooled effect size, the analysis helps identify whether any particular study disproportionately impacts the results, ensuring that the conclusions drawn are not unduly influenced by any single study and that the results are indeed consistent and reliable.

The possibility of publication bias in the meta-analysis was investigated using Egger’s linear regression test, Begg and Mazumdar’s rank correlation test, and funnel plots. The statistical significance level was defined as *p* < 0.05 for all outcomes. The Review Manager (v.5.4, Copenhagen, Denmark) (18) and ProMeta-3^®^ (19) meta-analysis software packages were used to perform all analyses in our study.

## 3 Results

**Figure 1:**
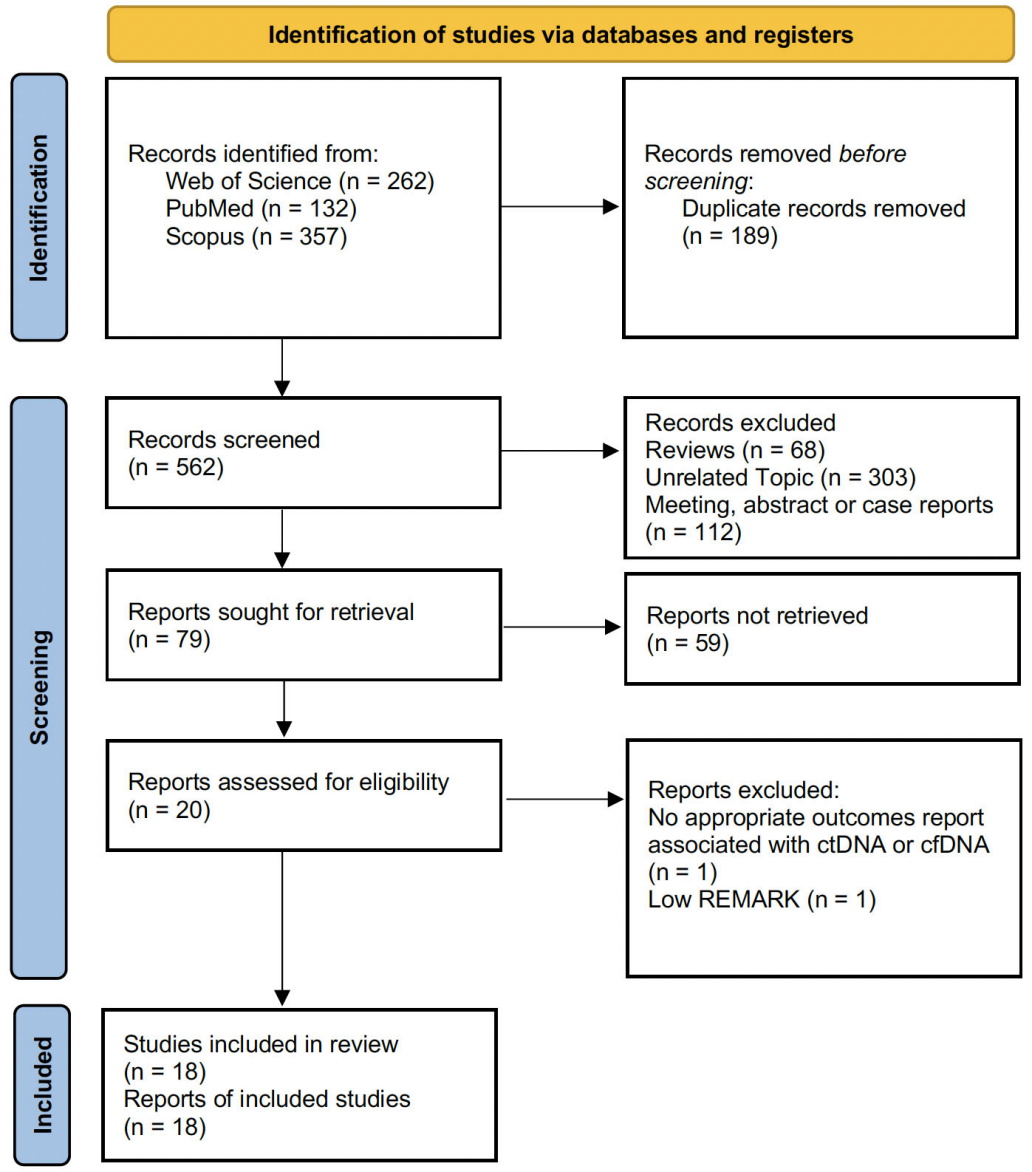
PRISMA flow diagram showing the number of records identified, included, and excluded, as well as the reasons for exclusions.

A total of 751 papers were identified through structured literature searches of the PubMed/Medline (n = 132), Scopus (n = 357), and WoS (n = 262) databases. After eliminating irrelevant and duplicate records, the full texts of the remaining 79 studies were meticulously examined in detail. A total of 20 studies were evaluated for further analysis. After detailed quality assessment, one study was excluded from the meta-analysis because of an insufficient quality score (REMARK score < 5.5) (20). After all the reviews were completed, a total of 18 articles (10–12, 21–35) that met the criteria for the current meta-analysis were included in the study. The PRISMA flowchart, which maps out the number of records identified, included, and excluded, as well as the reasons for exclusions, is presented visually in **Figure 1**.

### 3.1 Baseline characteristics of the primary studies

A total of 2,310 patients from 18 published papers (10–12, 21–35) were included in this meta-analysis. The sample sizes in the relevant studies ranged from 14 to 437. The main characteristics of the included studies are available in **Table 1**. The prognostic role of ctDNA in OS information was included in seven (12, 22, 25–27, 31, 33) of eighteen studies. The publication years of the studies included in the meta-analysis changed between 2002 and 2022. KRAS mutations were detected in almost all of the studies.

**Table 1:** Baseline characteristics of the studies included in the meta-analysis.

First author	Study year	Outcome	Sample size (n)	Characteristics	Gene of interest	ctDNA, cfDNA positivity (n)	Endpoints of meta-analysis
Chen et al. (21)	2010	Diagnostic Prognostic	91	Stage III n=29 Stage IV n=62	KRAS G12D, G12R, G12V, G12S, G12C, G12A and G13D KRAS codon 12	30/91	ctDNA
Däbritz et al. (11)	2009	Diagnostic	56	Irresectable n=23 Resectable n=25 Unknown n=8	KRAS codon 12	20/56	ctDNA
Hadano et al. (22)	2016	Diagnostic Prognostic	105	Stage I/II n=84 Stage III/IV n=21	KRAS	33/105	ctDNA, overall survival (HR=3.2, 95% CI=1.8–5.4, p<0.001)
Maire et al. (23)	2002	Diagnostic	47	Stage I n=5 Stage II/III n=19 Stage IV n=23	KRAS2 G12D	22/47	ctDNA
Le Calvez-Kelm et al. (24)	2016	Diagnostic	437	Local n=39 Regional n =143 Systemic n= 135 Unknown n =120	KRAS codons 12,13, 61	92/437	ctDNA
Pietrasz et al. (25)	2017	Diagnostic Prognostic Predictive	135	Resectable n=31 Locally advanced n=36 Metastatic n=68	Ion Colon and Lung panel	56/135	ctDNA, Overall survival (HR=1.96, 95% CI=1.20–3.20, p=0.007)
Tjensvoll et al. (27)	2016	Prognostic Predictive	14	Stage IV n=18 Advanced/metastatic	KRAS codon 12	10/14	ctDNA, overall survival (HR=1.31, p=0.047)
Chen et al. (12)	2017	Diagnostic Prognostic	189	Stage III n=40 Stage IV n=149	KRAS G12A, G12C, G12D, G12R, G12S, G12V, G13D	177/189	ctDNA, overall survival (HR=1.45, 95% CI=1.17–1.80, p=0.0007)
Cohen et al. (10)	2017	Diagnostic	221	Stage I n=19 Stage II n=192	KRAS	66/221	ctDNA
Del Re et al. (28)	2017	Diagnostic	27	Stage III n=4 Stage IV n=23	KRAS p.G12D, p.G12R, p.G12V, p.G13D	19/27	cfDNA
Huang et al. (29)	2022	Diagnostic	74	Stage III n=18 Stage IV n=56	KRAS G12D, G12R, G12V, G12C	26/35	cfDNA
Kim et al. (30)	2018	Diagnostic	106	Resectable n=41 Locally advanced n=25 Metastatic n=40	KRAS	53/106	cfDNA
Lin et al. (31)	2018	Diagnostic Prognostic	65	Stage I/II n=5 Stage III/IV n=60	KRAS G12V, G12D, G12R	20/65	ctDNA, overall survival (HR=3.1, 95% CI=1.6–4.9, p<0.001)
Pietrasz et al. (26)	2022	Diagnostic Prognostic	354	Metastatic n=255	KRAS, TP53 SMAD4	145/255	ctDNA, overall survival (HR=1.62, 95% CI=1.05–2.5, p=0.029)
Strijker et al. (32)	2020	Diagnostic	58	Metastatic n=58	KRAS, TP53, SMAD4, BRAF	26/58	ctDNA
Uesato et al. (33)	2020	Diagnostic Prognostic	104	Liver metastatic n=104	TP53, KRAS, APC, FBXW7, GNAS, ERBB2, CTNNB1, MAP2K1, EGFR, SMAD4, PIK3CA, BRAF, NRAS	52/104	ctDNA, overall survival (HR=3.1, 95% CI=1.9–5.0, p<0.001)
Wang et al. (34)	2022	Diagnostic	149	Stage I/II n=14 Stage III/IV n=91 Nonmalignant pancreatic massesn n=44	KRAS codon 12, G12D, G12R, G12V	37/105	ctDNA
Watanabe et al. (35)	2019	Diagnostic	78	Underwent surgery: IA/IB/IIA n=14 IIB/III/IV n=25; Did not undergo surgery: Stage III n=13 Stage IV n=22	KRAS codon 12, G12D, G12R, G12V	19/78	ctDNA

HR, hazard ratio; CI, confidence interval; N/A, not available.

In 18 included studies, the REMARK criteria (16) were used to assess the study quality. Overall, the studies included in the meta-analysis were of high quality (≥5.5). **Table 2** also illustrates the methodological quality evaluation of the included studies in detail.

**Table 2:** Quality assessment of the included studies according to the adapted REMARK criteria.

First author/year	C1	C2	C3	C4	C5	C6	C7	Total
Chen et al., 2010 (21)	1	1	1	1	1	1	1	7
Däbritz et al., 2009 (11)	1	1	0.5	1	1	1	1	6.5
Hadano et al., 2016 (22)	1	1	1	1	1	0.5	1	6.5
Maire et al., 2002 (23)	1	1	1	0.5	1	1	1	6.5
Le Calvez-Kelm et al., 2016 (24)	1	1	0.5	0.5	1	1	1	6
Pietrasz et al., 2017 (25)	1	1	1	1	1	1	1	7
Tjensvoll et al., 2016 (27)	1	1	1	1	1	1	1	7
Chen et al., 2017 (12)	1	1	1	1	0.5	1	1	6.5
Cohen et al., 2017 (10)	1	1	0.5	1	1	1	1	6.5
Del Re et al., 2017 (28)	1	1	0.5	1	1	1	1	7
Huang et al., 2022 (29)	1	1	0.5	0.5	1	1	1	6
Kim et al., 2018 (30)	1	1	1	1	1	0.5	1	6.5
Lin et al., 2018 (31)	1	1	1	0.5	1	1	1	6.5
Pietrasz et al., 2022 (26)	1	1	1	1	1	1	1	7
Strijker et al., 2020 (32)	1	1	1	0.5	0.5	1	1	6
Uesato et al., 2020 (33)	1	1	1	1	1	1	1	7
Wang et al., 2022 (34)	1	1	1	1	1	0.5	1	6.5
Watanabe et al., 2019 (35)	1	1	0.5	1	1	1	1	6.5

A maximum of 7 points could be allocated, and a minimum of 5.5 points had to be reached for inclusion. Green color is full score. Yellow color means half point.

### 3.2 Outcomes of the meta-analysis

#### 3.2.1 Meta-analysis of the diagnostic accuracy of ctDNA/cfDNA compared to that of CA 19-9 in all disease stages of pancreatic malignancies

**Figure 2:**
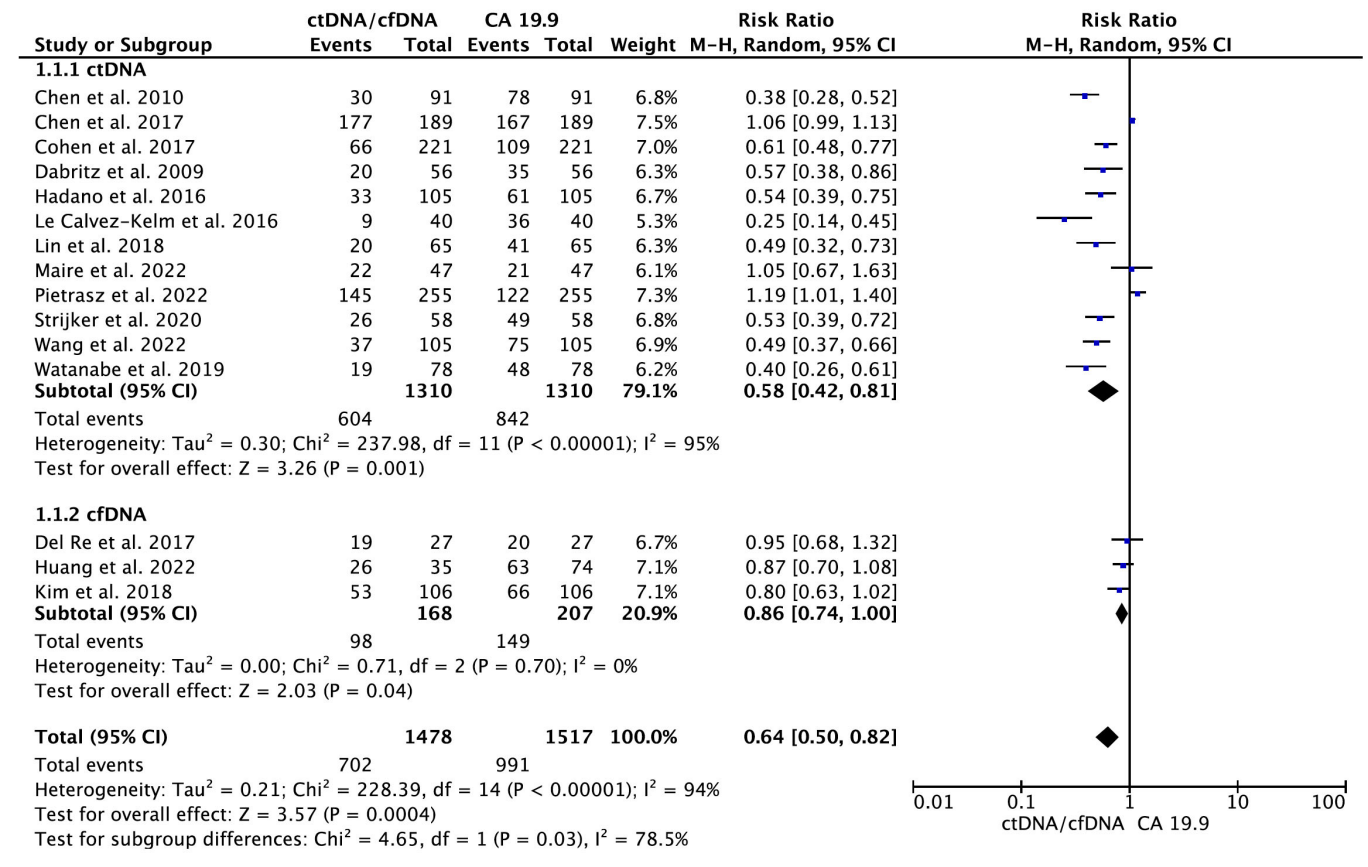
Meta-analysis of the diagnostic accuracy of circulating tumor DNA (ctDNA)/cell-free DNA (cfDNA) compared to CA 19-9 in all disease stages of pancreatic cancer.

A total of 15 eligible studies (16–20, 22, 24–30, 32, 33) containing data on CA 19.9 and the diagnostic accuracy of ctDNA/cfDNA were included in the primary meta-analysis. As shown in **Figure 2**, the results of the pooled meta-analysis confirmed that CA19–9 provides greater diagnostic accuracy than KRAS ctDNA at all disease stages (RR = 0.58, 95% CI = 0.42–0.81, *p* = 0.001). Significant heterogeneity was found between studies (I^2^ = 95%, *p <* 0.001). Therefore, the analysis was carried out using the random-effects model. Similarly, a pooled analysis of three primary studies indicated that CA19–9 provided significantly greater diagnostic accuracy than cfDNA at all disease stages (RR = 0.86, 95% CI = 0.74–1.00, *p* = 0.04). No significant publication bias was detected between studies (Egger’s test: *p* > 0.05; Begg’s test: *p* > 0.05) (**Supplementary Figure S1**).

#### 3.2.2 Meta-analysis of ctDNA in terms of its prognostic role in overall survival

**Figure 3:**
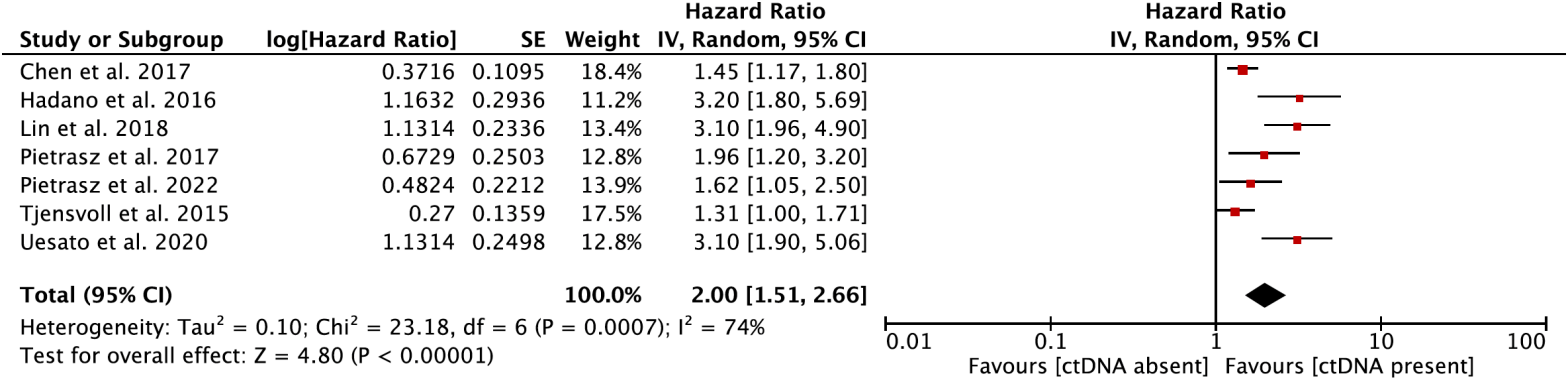
Forest plot showing the effect size of a meta-analysis evaluating the prognostic role of circulating tumor DNA (ctDNA) on overall survival.

In the secondary meta-analysis, data from a total of seven eligible papers (12, 22, 25–27, 31, 33) on the prognostic role of ctDNA in OS were evaluated. In the pooled analysis, ctDNA-positive patients had a worse OS (HR = 2.00, 95% CI = 1.51–2.66, *p <* 0.001) (**Figure 3**). Significant heterogeneity was observed among the included studies. Hence, the meta-analysis was carried out using the random-effects model (I^2^ = 74%, *p <* 0.001) (**Supplementary Figure S2**).

#### 3.2.3 Sensitivity analyses

To evaluate the robustness of our meta-analysis findings, sensitivity analyses were performed by systematically removing each study from the overall pool and observing the impact on the effect size (ES). The consistent ES across these recalculations underscores the reliability of the overall conclusions, as demonstrated in **Supplementary Figures S3** and **S4**.

## 4 Discussion

This current meta-analysis provides a comprehensive evaluation of ctDNA and cfDNA as potential diagnostic and prognostic biomarkers for pancreatic malignancies. Hence, this study provides a comprehensive perspective on research suggesting that liquid biopsies may be a potential instrument for the diagnosis and prognosis of pancreatic malignancies.

Reports of previous studies evaluating ctDNA and CA 19.9 for diagnostic accuracy have provided conflicting reports on the topic. In a study conducted by Chen et al., it was reported that the diagnostic accuracy of CA 19.9 was greater than that of ctDNA (21). Similarly, other studies conducted with various sample sizes have also reported that CA 19.9 has significantly greater diagnostic accuracy than ctDNA (10, 11, 32). However, several studies have proposed that the diagnostic accuracy of ctDNA is greater than that of CA 19.9 or that there is no significant difference between them (12, 13, 26). The observed discrepancy may be attributed to varying ctDNA detection techniques and patient cohorts. In the present meta-analysis, 12 primary studies provided information on the diagnostic accuracy of ctDNA against CA 19-9 (10–12, 21–27, 31–35). The detailed analysis revealed that CA19–9 offers greater diagnostic accuracy than ctDNA across all disease stages. Although the diagnostic accuracy of ctDNA is lower than that of the CA19–9 protein biomarker, the development of high-sensitivity and multigene NGS techniques is important for diagnosing several cancers, including pancreatic malignancies. It is critical to highlight that CA19–9 analysis has now become complementary to ctDNA analysis. The integration of both biomarkers in diagnostic and prognostic assessments offers a more comprehensive understanding of pancreatic cancer progression. While ctDNA provides real-time insights into tumor dynamics and genetic alterations, CA19–9 serves as an established marker that can enhance diagnostic accuracy when used alongside ctDNA. Taken together, these biomarkers should be considered for monitoring treatment response, detecting early relapses, and developing personalized treatment strategies, especially when surgical resection is not an option. In terms of cfDNA, reports from a total of three studies (28–30) were obtained in our study. The diagnostic accuracy of cfDNA was significantly lower than that of CA19.9 in the pooled analysis. However, the number of studies included in the meta-analysis was relatively small, and there were few statistical analyses. Therefore, it is essential to conduct more studies with larger sample sizes focusing on cfDNA to better understand its potential role in diagnosing pancreatic cancer.

Various studies have investigated the prognostic value of ctDNA in patients with early-stage or advanced-stage pancreatic cancer. In a study focusing on patients with resectable and detectable ctDNA, patients who were ctDNA-positive had a significantly worse prognosis (OS: 13.6 months) than those who were ctDNA-negative (OS: 27.6 months) (22). Similarly, in another study conducted by Chen et al., the K-ras mutation found in plasma DNA served as a predictive biomarker for poor prognosis in patients with unresectable pancreatic cancer (21). These findings suggest that it could serve as a valuable tool for determining the prognosis and developing personalized treatment strategies in patients with pancreatic cancer who cannot undergo surgical removal. In contrast to prior studies, another study indicated that the presence of ctDNA was not significantly associated with survival outcomes in a cohort of pancreatic cancer patients at all disease stages (36). This dissimilarity underlines the variability and complexity in the prognostic value of ctDNA across different patient populations and disease stages. The findings also suggest that the role of ctDNA in prognosis may be influenced by various factors, such as cancer stage, treatment methods, and individual patient characteristics. In this study, patients who were ctDNA positive were found to have poorer OS. Our meta-analysis highlights the prognostic clinical value of ctDNA in pancreatic malignancies, increasing the evidence level of existing prior studies. Therefore, these findings could have significant implications for patient management, influence treatment strategies, potentially guide treatment decisions, and enable more personalized therapeutic approaches for ctDNA-positive patients.

The potential role of ctDNA in screening and diagnosing malignant diseases remains unclear. Notably, the current situation in ctDNA parallels concerns regarding the use of CA19–9, another biomarker, which is a screening test for cancer according to international guidelines (37). There are several challenges to the widespread adoption of ctDNA. These challenges are related to the specificity, sensitivity, and variability of ctDNA levels across different cancers and cancer stages. Additionally, cost-effectiveness, ethical considerations, and the need for a comprehensive understanding of its implications across various types of malignancies contribute to the ongoing discussion. However, CA19–9 has limited application due to similar issues. Therefore, the future role of ctDNA in early cancer detection and screening has yet to be determined, pending further research and consensus in the scientific community.

Our meta-analysis had several limitations that should be discussed and highlighted. In this meta-analysis, a substantial amount of heterogeneity was observed, which can be attributed to large variations in the study populations and the methods used for detecting ctDNA. This variability may affect the consistency and comparability of the results, making it difficult to draw definitive conclusions from the pooled data. We utilized a random effects model in our statistical analyses to accommodate the variations among different patient groups. However, this approach does not eliminate the potential bias introduced by the diverse study populations. In particular, the limited number of studies examining the association between ctDNA and OS limits the generalizability of the results. This limited number of studies also restricted our capacity to conduct subgroup analyses, which could have provided deeper insights into the effects of ctDNA across different patient subpopulations or stages of the disease. Future studies with larger sample sizes and more homogeneous patient groups are needed to verify the findings of this meta-analysis and refine the understanding of the prognostic value of ctDNA in pancreatic cancer patients.

Moreover, the detection rate of ctDNA is affected by several uncertain factors, such as the tumor’s ability to release ctDNA into the bloodstream. This capability varies with the tumor’s size, type, stage, metabolic activity, and surrounding tissue environment. Another crucial factor is the rate at which ctDNA is cleared from the blood. This process is influenced by physiological factors such as degradation by nucleases and removal by organs such as the liver and kidneys. These variables contribute to the observed differences in ctDNA levels among patients and can significantly impact biomarker sensitivity and specificity in cancer detection and monitoring. Hence, the need for further understanding of these biological mechanisms in the context of this topic should be highlighted.

Despite these limitations, pooling data from primary studies has significantly increased the level of evidence. Therefore, this paper provides a strong perspective for future studies.

## 5 Conclusions

Taken together, the findings of this meta-analysis indicate that CA19.9 still provides greater diagnostic accuracy across all disease stages than KRAS mutations in ctDNA or cfDNA. However, the presence of detectable levels of ctDNA was associated with worse patient outcomes regarding OS in pancreatic malignancies. Recognizing the prognostic significance of ctDNA could significantly influence treatment decisions, enabling healthcare providers to tailor more personalized and effective therapeutic approaches. As the field of oncology continues to evolve, the role of these biomarkers in improving diagnosis, prognosis, and treatment monitoring may become more important. It is highly recommended that future research continue to investigate the potential role of ctDNA and cfDNA as biomarkers in larger samples and multicenter studies.

## Data availability statement

The raw data supporting the conclusions of this article will be made available by the authors, without undue reservation.

## Author contributions

MA: Conceptualization, Data curation, Formal analysis, Funding acquisition, Investigation, Methodology, Project administration, Resources, Software, Supervision, Validation, Visualization, Writing – original draft, Writing – review & editing. Aİ: Data curation, Investigation, Resources, Validation, Visualization, Writing – original draft, Writing – review & editing. YB: Conceptualization, Investigation, Methodology, Project administration, Resources, Software, Supervision, Validation, Writing – original draft, Writing – review & editing. NO: Conceptualization, Data curation, Funding acquisition, Investigation, Methodology, Project administration, Software, Supervision, Validation, Visualization, Writing – original draft, Writing – review & editing.
