# Supplementary material for: Evaluation of the diagnostic and prognostic clinical values of circulating tumor DNA and cell-free DNA in pancreatic malignancies: a comprehensive meta-analysis
Source: Front Oncol. 2024 Jun 25;14:1382369. doi: 10.3389/fonc.2024.1382369 (PMC11231086; doi:10.3389/fonc.2024.1382369)
Supplement: Supplementary file 1 [file DataSheet_1.pdf]

Supplementary appendix

Table S1 The PRISMA checklist

Section and Topic	Item #	Checklist item	Location where item is reported
**TITLE**			
Title	1	Identify the report as a systematic review.	1
**ABSTRACT**			
Abstract	2	See the PRISMA 2020 for Abstracts checklist.	2
**INTRODUCTION**			
Rationale	3	Describe the rationale for the review in the context of existing knowledge.	3
Objectives	4	Provide an explicit statement of the objective(s) or question(s) the review addresses.	3
**METHODS**			
Eligibility criteria	5	Specify the inclusion and exclusion criteria for the review and how studies were grouped for the syntheses.	4
Information sources	6	Specify all databases, registers, websites, organisations, reference lists and other sources searched or consulted to identify studies. Specify the date when each source was last searched or consulted.	4
Search strategy	7	Present the full search strategies for all databases, registers and websites, including any filters and limits used.	4
Selection process	8	Specify the methods used to decide whether a study met the inclusion criteria of the review, including how many reviewers screened each record and each report retrieved, whether they worked independently, and if applicable, details of automation tools used in the process.	5
Data collection process	9	Specify the methods used to collect data from reports, including how many reviewers collected data from each report, whether they worked independently, any processes for obtaining or confirming data from study investigators, and if applicable, details of automation tools used in the process.	5
Data items	10a	List and define all outcomes for which data were sought. Specify whether all results that were compatible with each outcome domain in each study were sought (e.g. for all measures, time points, analyses), and if not, the methods used to decide which results to collect.	5
	10b	List and define all other variables for which data were sought (e.g. participant and intervention characteristics, funding sources). Describe any assumptions made about any missing or unclear information.	6
Study risk of bias assessment	11	Specify the methods used to assess risk of bias in the included studies, including details of the tool(s) used, how many reviewers assessed each study and whether they worked independently, and if applicable, details of automation tools used in the process.	6
Effect measures	12	Specify for each outcome the effect measure(s) (e.g. risk ratio, mean difference) used in the synthesis or presentation of results.	6
Synthesis methods	13a	Describe the processes used to decide which studies were eligible for each synthesis (e.g. tabulating the study intervention characteristics and comparing against the planned groups for each synthesis (item #5)).	6-7
	13b	Describe any methods required to prepare the data for presentation or synthesis, such as handling of missing summary statistics, or data conversions.	6-7
	13c	Describe any methods used to tabulate or visually display results of individual studies and syntheses.	6-7
	13d	Describe any methods used to synthesize results and provide a rationale	6-7

Section and Topic	Item #	Checklist item	Location where item is reported
		for the choice(s). If meta-analysis was performed, describe the model(s), method(s) to identify the presence and extent of statistical heterogeneity, and software package(s) used.	
	13e	Describe any methods used to explore possible causes of heterogeneity among study results (e.g. subgroup analysis, meta-regression).	6-7
	13f	Describe any sensitivity analyses conducted to assess robustness of the synthesized results.	6-7
Reporting bias assessment	14	Describe any methods used to assess risk of bias due to missing results in a synthesis (arising from reporting biases).	6-7
Certainty assessment	15	Describe any methods used to assess certainty (or confidence) in the body of evidence for an outcome.	6-7
**RESULTS**			
Study selection	16a	Describe the results of the search and selection process, from the number of records identified in the search to the number of studies included in the review, ideally using a flow diagram.	7
	16b	Cite studies that might appear to meet the inclusion criteria, but which were excluded, and explain why they were excluded.	7
Study characteristics	17	Cite each included study and present its characteristics.	8
Risk of bias in studies	18	Present assessments of risk of bias for each included study.	7-8
Results of individual studies	19	For all outcomes, present, for each study: (a) summary statistics for each group (where appropriate) and (b) an effect estimate and its precision (e.g. confidence/credible interval), ideally using structured tables or plots.	8-9
Results of syntheses	20a	For each synthesis, briefly summarise the characteristics and risk of bias among contributing studies.	8-9
	20b	Present results of all statistical syntheses conducted. If meta-analysis was done, present for each the summary estimate and its precision (e.g. confidence/credible interval) and measures of statistical heterogeneity. If comparing groups, describe the direction of the effect.	8-9
	20c	Present results of all investigations of possible causes of heterogeneity among study results.	9
	20d	Present results of all sensitivity analyses conducted to assess the robustness of the synthesized results.	9-10
Reporting biases	21	Present assessments of risk of bias due to missing results (arising from reporting biases) for each synthesis assessed.	9-10
Certainty of evidence	22	Present assessments of certainty (or confidence) in the body of evidence for each outcome assessed.	9-10
**DISCUSSION**			
Discussion	23a	Provide a general interpretation of the results in the context of other evidence.	10
	23b	Discuss any limitations of the evidence included in the review.	11-12
	23c	Discuss any limitations of the review processes used.	11-12
	23d	Discuss implications of the results for practice, policy, and future research.	11-12
**OTHER INFORMATION**			
Registration and protocol	24a	Provide registration information for the review, including register name and registration number, or state that the review was not registered.	3
	24b	Indicate where the review protocol can be accessed, or state that a protocol was not prepared.	3
	24c	Describe and explain any amendments to information provided at registration or in the protocol.	3

Section and Topic	Item #	Checklist item	Location where item is reported
Support	25	Describe sources of financial or non-financial support for the review, and the role of the funders or sponsors in the review.	2
Competing interests	26	Declare any competing interests of review authors.	2
Availability of data, code and other materials	27	Report which of the following are publicly available and where they can be found: template data collection forms; data extracted from included studies; data used for all analyses; analytic code; any other materials used in the review.	2

Table S2 Search strategy used for the meta-analysis in the PubMed/Medline, Web of Science, and Scopus databases

**Database**	**Keywords**
**Pubmed/Medline**	
#1	Search: **Pancreatic[Title/Abstract]** Sort by: **Most Recent**<br>"Pancreatic"[Title/Abstract]
#2	Search: **((((Carcinoma[Title/Abstract]) OR (cancer[Title/Abstract])) OR (neoplasm[Title/Abstract])) OR (tumor[Title/Abstract])) OR (tumour[Title/Abstract])** Sort by: **Most Recent**<br>"Carcinoma"[Title/Abstract] OR "cancer"[Title/Abstract] OR "neoplasm"[Title/Abstract] OR "tumor"[Title/Abstract] OR "tumour"[Title/Abstract]
#3	Search: **(circulating cfDNA[Title/Abstract]) OR (circulating ctDNA[Title/Abstract])** Sort by: **Most Recent**<br>"circulating cfdna"[Title/Abstract] OR (("blood circulation"[MeSH Terms] OR ("blood"[All Fields] AND "circulation"[All Fields]) OR "blood circulation"[All Fields] OR "circulation"[All Fields] OR "circulations"[All Fields] OR "circulate"[All Fields] OR "circulated"[All Fields] OR "circulates"[All Fields] OR "circulating"[All Fields]) AND "ctDNA"[Title/Abstract])<br>**Translations**<br>**circulating:** "blood circulation"[MeSH Terms] OR ("blood"[All Fields] AND "circulation"[All Fields]) OR "blood circulation"[All Fields] OR "circulation"[All Fields] OR "circulations"[All Fields] OR "circulate"[All Fields] OR "circulated"[All Fields] OR "circulates"[All Fields] OR "circulating"[All Fields]
#4	**#1 AND #2 AND #3**<br><br>**Search: ((Pancreatic[Title/Abstract]) AND (((((Carcinoma[Title/Abstract]) OR (cancer[Title/Abstract])) OR (neoplasm[Title/Abstract])) OR (tumor[Title/Abstract])) OR**

	**(tumour[Title/Abstract]))) AND ((circulating cfDNA[Title/Abstract]) OR (circulating ctDNA[Title/Abstract])) Sort by: Most Recent**<br>"Pancreatic"[Title/Abstract] AND ("Carcinoma"[Title/Abstract] OR "cancer"[Title/Abstract] OR "neoplasm"[Title/Abstract] OR "tumor"[Title/Abstract] OR "tumour"[Title/Abstract]) AND ("circulating cfdna"[Title/Abstract] OR (("blood circulation"[MeSH Terms] OR ("blood"[All Fields] AND "circulation"[All Fields]) OR "blood circulation"[All Fields] OR "circulation"[All Fields] OR "circulations"[All Fields] OR "circulate"[All Fields] OR "circulated"[All Fields] OR "circulates"[All Fields] OR "circulating"[All Fields]) AND "ctDNA"[Title/Abstract]))<br>Translations<br>circulating: "blood circulation"[MeSH Terms] OR ("blood"[All Fields] AND "circulation"[All Fields]) OR "blood circulation"[All Fields] OR "circulation"[All Fields] OR "circulations"[All Fields] OR "circulate"[All Fields] OR "circulated"[All Fields] OR "circulates"[All Fields] OR "circulating"[All Fields]
**Web of Science (WoS)**	
#1	TOPIC:<br>(ALL=(Pancreatic)) Indexes=SCI-EXPANDED, SSCI, A&HCI, CPCI-S, CPCI-SSH, BKCIS, BKCI-SSH, ESCI, CCR-EXPANDED, IC Timespan=All years
#2	((((ALL=(Carcinoma)) OR ALL=(cancer)) OR ALL=(neoplasm)) OR ALL=(tumor)) OR ALL=(tumour) Indexes=SCI-EXPANDED, SSCI, A&HCI, CPCI-S, CPCI-SSH, BKCIS, BKCI-SSH, ESCI, CCR-EXPANDED, IC Timespan=All years
#3	(ALL=(circulating cfDNA)) OR ALL=(circulating ctDNA) Indexes=SCI-EXPANDED, SSCI, A&HCI, CPCI-S, CPCI-SSH, BKCIS, BKCI-SSH, ESCI, CCR-EXPANDED, IC Timespan=All years

#4	#1 AND #2 AND #3 Indexes=SCI-EXPANDED, SSCI, A&HCI, CPCI-S, CPCI-SSH, BKCIS, BKCI-SSH, ESCI, CCR-EXPANDED, IC Timespan=All years
**Scopus**	
#1	pancreatic
#2	carcinoma
#3	cancer
#4	neoplasm
#5	tumor*
#6	tumour*
#7	#2 OR #3 OR #4 OR #5 OR #6
#8	circulating cfDNA
#9	circulating ctDNA
#10	#8 OR #9
#11	#1 AND #7 AND #10

Table S3 The adapted version of the REporting recommendations for tumor MARKer prognostic studies (REMARK) criteria for biomarker studies (McShane et al. 2005)

**Adapted REMARK criteria for quality assessment (1 point/criteria)**	
**1**	Case selection adequate (baselines from medical chart)
**2**	State the marker examined and the aim of the study
**3**	Reporting at least the following characteristics: location of primary tumor (esophagus, stomach or pancreas), disease stage, histology and received treatment
**4**	State the time and type of sampling (serum/plasma)
**5**	State the assay methods used and provide a detailed protocol (at least cfDNA isolation, sequence method and sequence depth)
**6**	A clear description of the flow of patients through the study
**7**	A clear description of the reasons of dropout

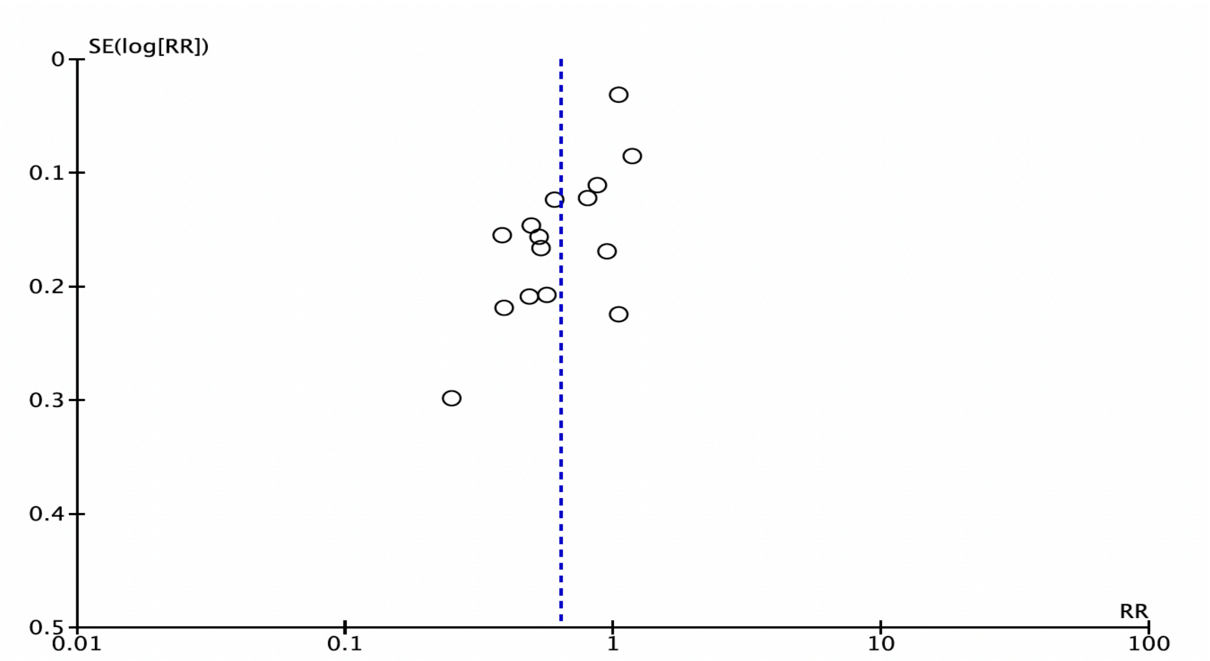

Fig. S1 The funnel plot of the meta-analysis of diagnostic accuracy of ctDNA compared to CA 19.9 in all disease stages of pancreatic cancer is approximately symmetrical and, in accordance with the results of Egger's ($p > .05$) and Begg's ($p > .05$) tests, fades the possibility of potential publication bias

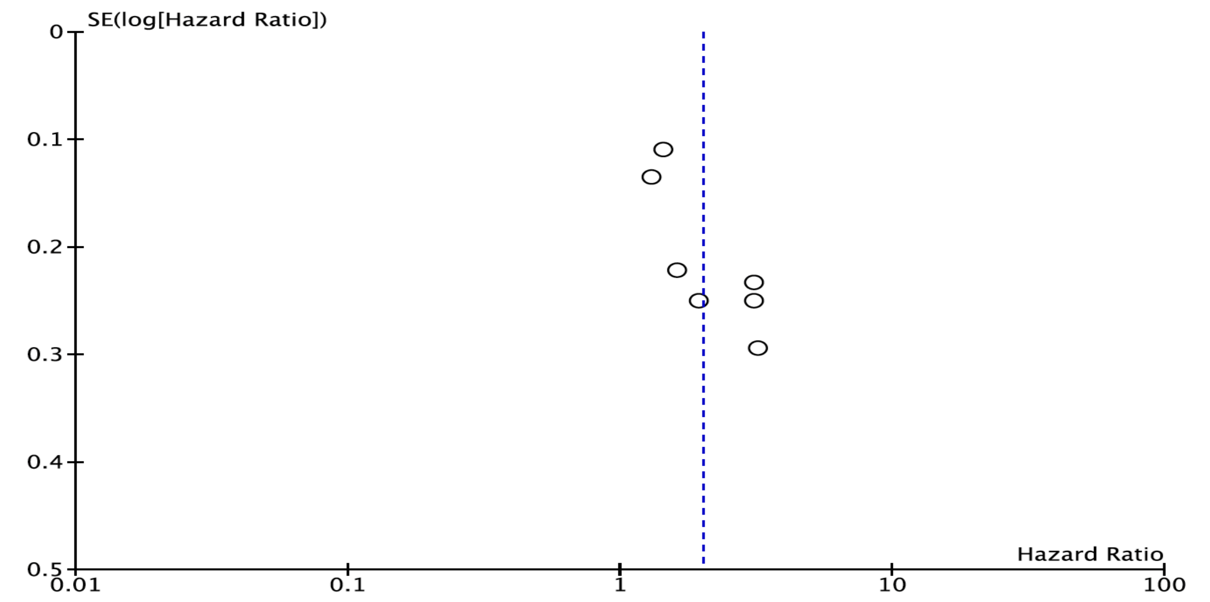

Fig. S2 The funnel plot of the meta-analysis of ctDNA in terms of the prognostic role on overall survival is approximately symmetrical and, in accordance with the results of Egger's ($P > .05$) and Begg's ($P > .05$) tests, fades the possibility of potential publication bias

	ES	95% CI	Sig.
Chen et al. 2010	0.67	0.55 / 0.82	0.000
Chen et al. 2017	0.61	0.49 / 0.77	0.000
Cohen et al. 2017	0.64	0.52 / 0.80	0.000
Dabritz et al. 2017	0.65	0.52 / 0.80	0.000
Del Re et al. 2017	0.62	0.50 / 0.78	0.000
Hadano et al. 2016	0.65	0.52 / 0.81	0.000
Huang et al. 2022	0.62	0.50 / 0.79	0.000
Kim et al. 2018	0.63	0.50 / 0.79	0.000
Le Calvez-Kelm et al. 2016	0.67	0.55 / 0.83	0.000
Lin et al. 2018	0.65	0.53 / 0.81	0.000
Maire et al. 2022	0.62	0.50 / 0.77	0.000
Pietrasz et al. 2022	0.61	0.48 / 0.77	0.000
Strijker et al. 2020	0.65	0.52 / 0.81	0.000
Wang et al. 2022	0.65	0.53 / 0.81	0.000
Watanabe et al. 2019	0.66	0.54 / 0.82	0.000

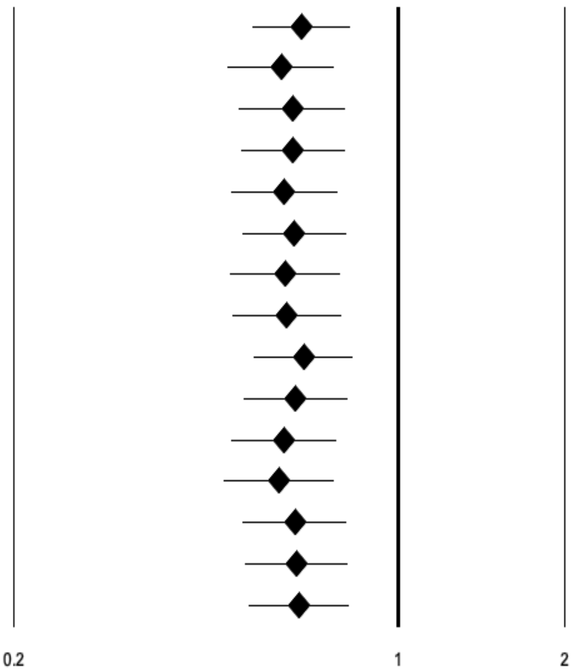

Fig. S3 Sensivity analyses of diagnostic accuracy of ctDNA/cfDNA compared to CA 19.9 in all disease stages of pancreatic cancer (ES = Risk raito; Sig = Statistical significance)

	ES	95% CI	Sig.
Chen et al. 2017	2.17	1.53 / 3.08	0.000
Hadano et al. 2016	1.88	1.41 / 2.50	0.000
Lin et al. 2018	1.85	1.41 / 2.44	0.000
Pietrasz et al. 2017	2.02	1.47 / 2.80	0.000
Pietrasz et al. 2022	2.09	1.50 / 2.91	0.000
Tjensvoll et al. 2015	2.20	1.59 / 3.04	0.000
Uesato et al. 2020	1.86	1.41 / 2.47	0.000

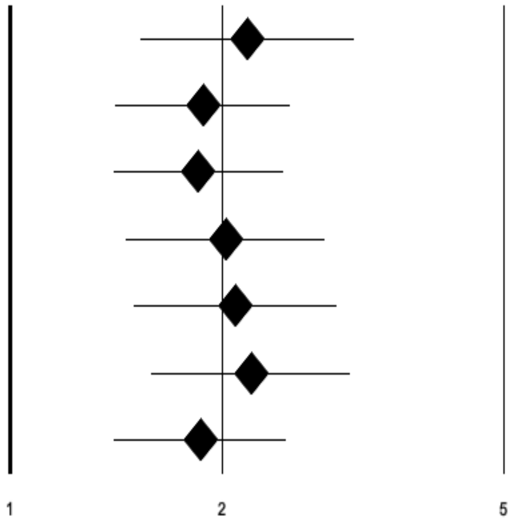

Fig. S4 Sensivity analysis of ctDNA in terms of its prognostic role in overall survival (ES = Risk raito; Sig = Statistical significance)

References

Chen H, Tu H, Meng ZQ, Chen Z, Wang P, Liu LM. K-ras mutational status predicts poor prognosis in unresectable pancreatic cancer. Eur J Surg Oncol. 2010;36(7):657-62. https://doi.org/10.1016/j.ejso.2010.05.014

Däbritz J, Preston R, Hänfler J, Oettle H. Follow-up study of K-ras mutations in the plasma of patients with pancreatic cancer: correlation with clinical features and carbohydrate antigen 19-9. Pancreas. 2009;38(5):534-41. https://doi.org/10.1097/MPA.0b013e31819f6376

Hadano N, Murakami Y, Uemura K, Hashimoto Y, Kondo N, Nakagawa N, et al. Prognostic value of circulating tumour DNA in patients undergoing curative resection for pancreatic cancer. Br J Cancer. 2016;115(1):59-65. https://doi.org/10.1038/bjc.2016.175

Maire F, Micard S, Hammel P, Voitot H, Lévy P, Cugnenc PH, Ruszniewski P, Puig PL. Differential diagnosis between chronic pancreatitis and pancreatic cancer: value of the detection of KRAS2 mutations in circulating DNA. Br J Cancer. 2002;87(5):551-4. https://doi.org/10.1038/sj.bjc.6600475

Le Calvez-Kelm F, Foll M, Wozniak MB, Delhomme TM, Durand G, Chopard P, et al. KRAS mutations in blood circulating cell-free DNA: a pancreatic cancer case-control. Oncotarget. 2016;7(48):78827-78840. https://doi.org/10.18632/oncotarget.12386

Pietrasz D, Pécuchet N, Garlan F, Didelot A, Dubreuil O, Doat S, et al. Plasma Circulating Tumor DNA in Pancreatic Cancer Patients Is a Prognostic Marker. Clin Cancer Res. 2017;23(1):116-123. https://doi.org/10.1158/1078-0432.CCR-16-0806

Tjensvoll K, Lapin M, Buhl T, Oltedal S, Steen-Ottosen Berry K, Gilje B, et al. Clinical relevance of circulating KRAS mutated DNA in plasma from patients with advanced pancreatic cancer. Mol Oncol. 2016;10(4):635-43. https://doi.org/10.1016/j.molonc.2015.11.012

Chen I, Raymond VM, Geis JA, Collisson EA, Jensen BV, Hermann KL, et al. Ultrasensitive plasma ctDNA *KRAS* assay for detection, prognosis, and assessment of therapeutic response in patients with unresectable pancreatic ductal adenocarcinoma. Oncotarget. 2017;8(58):97769-97786. https://doi.org/10.18632/oncotarget.22080

Cohen JD, Javed AA, Thoburn C, Wong F, Tie J, Gibbs P, et al. Combined circulating tumor DNA and protein biomarker-based liquid biopsy for the earlier detection of pancreatic cancers. Proc Natl Acad Sci U S A. 2017;114(38):10202-10207. https://doi.org/10.1073/pnas.1704961114

Del Re M, Vivaldi C, Rofi E, Vasile E, Miccoli M, Caparello C, et al. Early changes in plasma DNA levels of mutant KRAS as a sensitive marker of response to chemotherapy in pancreatic cancer. Sci Rep. 2017;7(1):7931. https://doi.org/10.1038/s41598-017-08297-z

Huang CJ, Huang WY, Chen CY, Chao YJ, Chiang NJ, Shan YS. Cancer-cell-derived cell-free DNA can predict distant metastasis earlier in pancreatic cancer: a prospective cohort study. Ther Adv Med Oncol. 2022;14:17588359221106558. https://doi.org/10.1177/17588359221106558

Kim MK, Woo SM, Park B, Yoon KA, Kim YH, Joo J, et al. Prognostic Implications of Multiplex Detection of *KRAS* Mutations in Cell-Free DNA from Patients with Pancreatic Ductal Adenocarcinoma. Clin Chem. 2018;64(4):726-734. https://doi.org/10.1373/clinchem.2017.283721

Lin M, Alnaggar M, Liang S, Chen J, Xu K, Dong S, et al. Circulating Tumor DNA as a Sensitive Marker in Patients Undergoing Irreversible Electroporation for Pancreatic Cancer. Cell Physiol Biochem. 2018;47(4):1556-1564. https://doi.org/10.1159/000490874

Pietrasz D, Wang-Renault S, Taieb J, Dahan L, Postel M, Durand-Labrunie J, et al. Prognostic value of circulating tumour DNA in metastatic pancreatic cancer patients: post-hoc analyses of two clinical trials. Br J Cancer. 2022;126(3):440-448. https://doi.org/10.1038/s41416-021-01624-2

Strijker M, Soer EC, de Pastena M, Creemers A, Balduzzi A, Beagan JJ, et al. Circulating tumor DNA quantity is related to tumor volume and both predict survival in metastatic pancreatic ductal adenocarcinoma. Int J Cancer. 2020;146(5):1445-1456. https://doi.org/10.1002/ijc.32586

Uesato Y, Sasahira N, Ozaka M, Sasaki T, Takatsuki M, Zembutsu H. Evaluation of circulating tumor DNA as a biomarker in pancreatic cancer with liver metastasis. PLoS One. 2020;15(7):e0235623. https://doi.org/10.1371/journal.pone.0235623

Wang R, Zhao Y, Wang Y, Zhao Z, Chen Q, Duan Y, et al. Diagnostic and Prognostic Values of KRAS Mutations on EUS-FNA Specimens and Circulating Tumor DNA in Patients With Pancreatic Cancer. Clin Transl Gastroenterol. 2022;13(5):e00487. https://doi.org/10.14309/ctg.0000000000000487

Watanabe F, Suzuki K, Tamaki S, Abe I, Endo Y, Takayama Y, et al. Longitudinal monitoring of KRAS-mutated circulating tumor DNA enables the prediction of prognosis and

therapeutic responses in patients with pancreatic cancer. PLoS One. 2019;14(12):e0227366. https://doi.org/10.1371/journal.pone.0227366
